# A case of pulmonary embolism with bad warfarin anticoagulant effects caused by *E. coli* infection

**DOI:** 10.1515/biol-2022-0539

**Published:** 2023-01-24

**Authors:** Weifeng Huang, Qingqing Cai, Yan Huo, Jin Tang, Yan Chen, Yuan Fang, Yihan Lu

**Affiliations:** Department of Intensive Care Medicine, Shanghai Sixth People’s Hospital Affiliated to Shanghai Jiao Tong University School of Medicine, Shanghai 200233, China; Genoxor Medical Science and Technology Inc., Shanghai 201112, China; Department of Pharmacy, Shanghai Sixth People’s Hospital Affiliated to Shanghai Jiao Tong University School of Medicine, Shanghai 200233, China; Department of Clinical Laboratory, Shanghai Sixth People’s Hospital Affiliated to Shanghai Jiao Tong University School of Medicine, Shanghai 200233, China; Department of Clinical Pharmacology, Shanghai Sixth People’s Hospital Affiliated to Shanghai Jiao Tong University School of Medicine, Shanghai 200233, China; Department of Epidemiology, Ministry of Education Key Laboratory of Public Health Safety, School of Public Health, Fudan University, No. 130, Dong’an Road, Shanghai 200032, China

**Keywords:** warfarin, anticoagulation, *Escherichia coli*, infection, metagenomic next-generation sequencing

## Abstract

Warfarin is an anticoagulant commonly used as an oral drug in preventing and treating thromboembolic diseases. The international normalized ratio (INR) is a clinical monitoring anticoagulation intensity index that adjusts the dose based on important references. In particular, INR value must be strictly monitored when warfarin is used for anticoagulation therapy in infected patients. Herein, we report a 54-year-old female patient diagnosed with pulmonary embolism and venous thrombosis of the lower limbs. After the warfarin administration, the INR was always substandard. The patient did not take other warfarin-interacting drugs or foods during the hospital stay. Metagenome next-generation sequencing suggested a bloodstream infection caused by *Escherichia coli*, which was further confirmed by blood culture. After meropenem administration for anti-infective treatment, the INR value rose rapidly to a standard level. Considering the lack of relevant reports, this case is the first report of potential interaction between *E. coli* and warfarin. Further, in patients with thromboembolic diseases complicated by infection, antibiotics should be chosen reasonably with close monitoring of the INR to avoid the interaction of warfarin and antibiotics and to ensure the effectiveness and safety of warfarin treatment.

## 1 Introduction

Pulmonary embolism is a frequent cardiovascular disease. It is mainly a pathological syndrome of respiratory dysfunction. Ninety percent of the emboli in acute pulmonary embolism are venous thrombosis of the lower limbs. Pulmonary infection is the most common complication of pulmonary embolism [1,2]. Warfarin is an oral drug commonly used for preventing and treating thromboembolic diseases. It acts as an anticoagulant by inhibiting the activity of vitamin K oxidoreductase (VKOR) and inhibiting the mutual conversion of vitamin K and 2,3-epoxides [3]. The treatment window of this drug is relatively narrow, and excessive use can easily cause bleeding and other adverse reactions. The anticoagulant effect of warfarin may be affected by anti-infective agents in patients with thromboembolism and infection, such as ciprofloxacin and miconazole are highly likely to increase the anticoagulant effect of warfarin, thereby increasing the international normalized ratio (INR) value and the risk of bleeding in patients [4,5]. On the contrary, griseofulvin, nafcillin, ribavirin, and rifampicin may inhibit the anticoagulant effects of warfarin [6,7].

The interaction of warfarin with other drugs has been studied extensively and showed that carbapenem antibiotics (meropenem, imipenem) have little effect on the anticoagulant effect of warfarin without liver enzyme metabolization. Therefore, carbapenem is the antibiotic of choice in infectious patients who need to use warfarin [8,9]. There is no interaction with warfarin reported for meropenem that was used in the present case. Simultaneously, considering the lack of relevant data on the interaction between microorganisms and warfarin, the possibility of interaction between *Escherichia coli* and warfarin was explored in this case study.

## 2 Case presentation

The patient, a 54-year-old female, admitted to the hospital on May 26, was diagnosed with pulmonary embolism and venous thrombosis of the lower limbs mainly due to sudden shortness of breath and chest tightness for 1 day while getting up. Physical examination of the patient upon admission revealed no abnormalities. Pulmonary artery computed tomography angiography showed extensive embolisms in each branch of the two pulmonary arteries. Lower extremity color doppler ultrasound showed hypoechoic muscular venous plexus in both legs and possible thrombosis.

**Figure 1:**
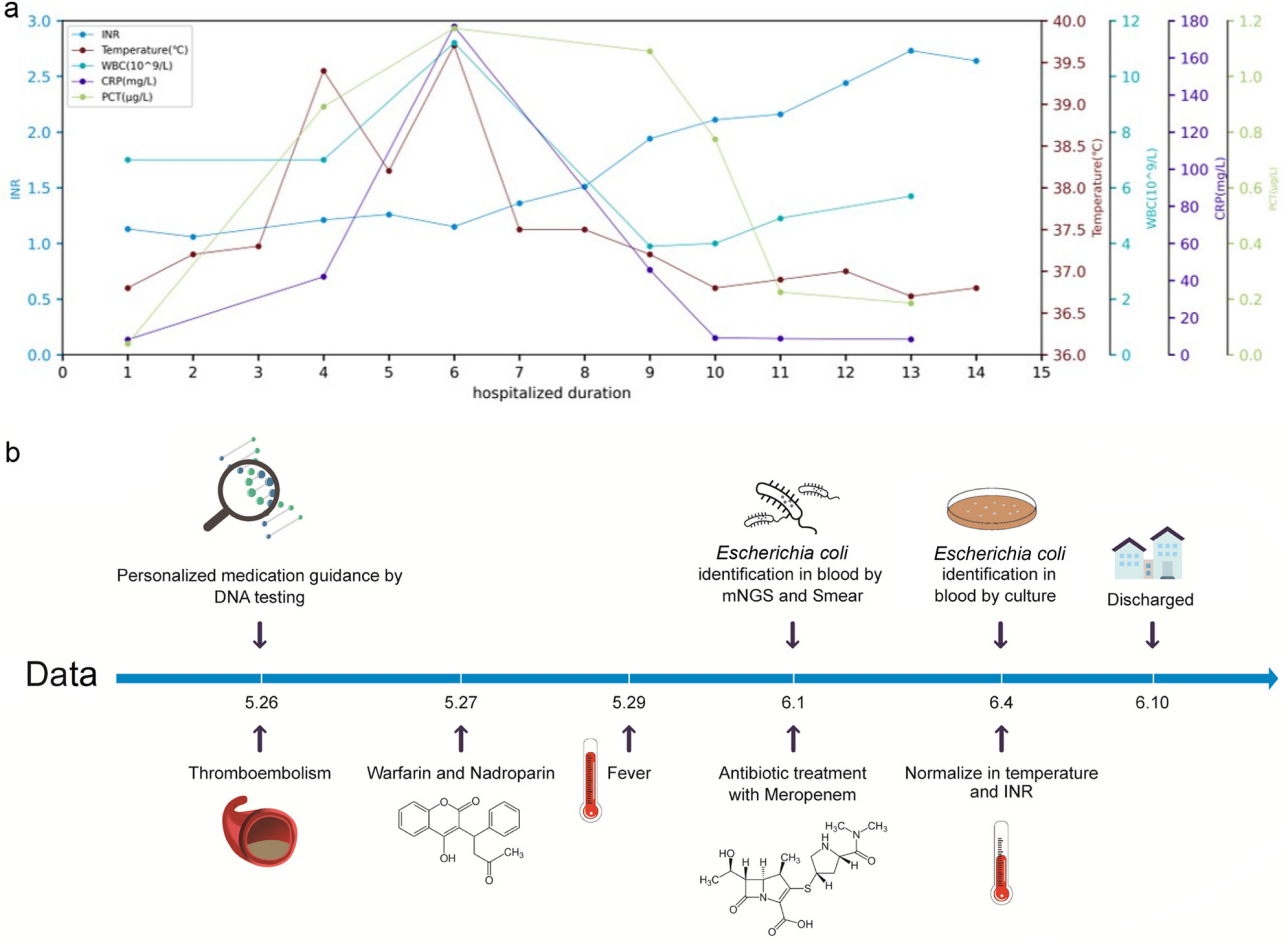
Timeline of this case and diagnostic workup. (a) The INR increases with the control of infection. (b) The timeline recorded essential events of the patient during hospitalization.

On admission, the patient was administered warfarin for thrombosis in the upper and lower pulmonary artery branches and between the calf muscles, and the INR was 1.13 (Figure 1). Meanwhile, as gene polymorphism significantly affects the dose of warfarin, among which *CYP2C9* (1075 A > C rs1057910) and *VKORC1* (1639G > A rs9923231) are the most significant [10,11], the key loci of *CYP2C9* and *VKORC1* genes were tested to guide warfarin administration. On the second day of admission (May 27), the INR was 1.06 (Figure 1), and the gene test results showed that the CYP2C9 activity of the patient was regular and the expression level of VKORC1 enzyme was decreased. According to the international warfarin pharmacogenomics consortium warfarin dose prediction model [12], we calculated the dose of warfarin, including load dose and maintenance dose: the patient was administered an initial 2-day dose of 6.25 mg, followed by a maintenance dose of 3.75 and 5.0 mg administered alternatively. Meanwhile, she was administered nadroparin 0.6 mL q12 h. In the following 2 days, the INR value of warfarin was still lower than 2.00, even after administering a higher dose of warfarin. Warfarin did not show the expected anticoagulant effect. To reduce the risk of bleeding and achieve an anticoagulant effect, the patient was administered nadroparin 0.6 mL q12 h and warfarin 5 mg q.d.

**Figure 2:**
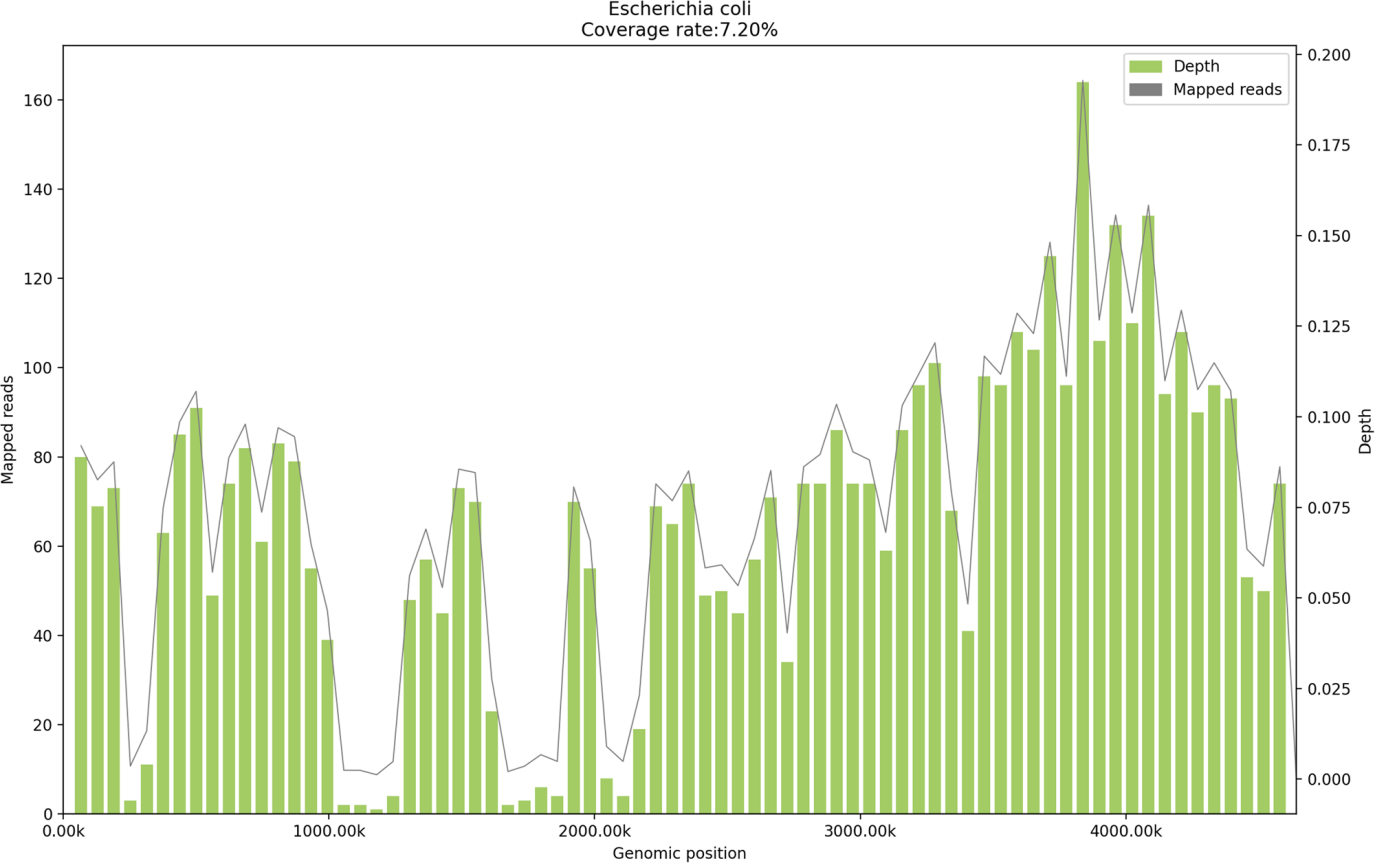
Data from mNGS. The location of the detected nucleic acid sequences of *E. coli* in blood, which yielded a total coverage of 7.2%.

Worse still, 2 days after admission (May 29), the patient had a fever with a body temperature of 39.4°C (Figure 1). Blood cell analysis on May 29 showed a neutrophil ratio (NEU%) of 75.7% and C-reactive protein at 42.1 mg/mL (Figure 1), indicating infection based on the hemogram. Therefore, vulvar secretions, urine, and blood samples were collected and cultured, but the test results were all negative. In the next few days, the patient had persistent fever, accompanied by a poor anticoagulant effect (INR < 2.00) (Figure 1). On May 31, routine blood testing showed that the percentage of human neutrophils increased to 83.5%, still indicating apparent infection. The culture smears of peripheral blood were then obtained for a second time and metagenomic next-generation sequencing (mNGS) was performed. *E. coli* was detected in the peripheral blood in mNGS (242 unique reads) (Figure 2). Then, meropenem (1 g q8 h) was administered as an empirical anti-infective therapy on June 1 for 4 days. *E. coli* was detected in the blood culture on June 4, and a bloodstream infection caused by *E. coli* was confirmed.

After starting meropenem treatment with warfarin, with the neutrophils and C-reactive protein levels of the patient returned to normal levels, the INR value of the patients also increased gradually from 1.36 (June 1) to 2.11, maintaining the best anticoagulant effect of warfarin on June 4 (Figure 1). After excluding the interaction between warfarin and the drugs or food used during hospitalization, interaction with *E. coli* infection was speculated to cause a decrease in the anticoagulant effect of warfarin, indicated by a reduction in the INR of patients.


**Informed consent:** Informed consent has been obtained from all individuals included in this study.
**Ethical approval:** The research related to human use has been complied with all the relevant national regulations, institutional policies and in accordance the tenets of the Helsinki Declaration, and has been approved by the authors’ institutional review board or equivalent committee.

## 3 Discussion

In this case, a 54-year-old female patient with pulmonary embolism and venous thrombosis of the lower limbs was treated with warfarin; while, at the recommended dose, the anticoagulant effect of warfarin was poor. During this period, the patient showed fever symptoms, and the mNGS and the blood culture results revealed *E. coli* bloodstream infection. Meropenem was immediately used as anti-infective treatment along with warfarin for 4 days. After starting meropenem, the INR increased from 1.36 to 2.11. The INR remained at approximately 2.00 after the infection index decreased and after stopping the meropenem used, indicating a time correlation between the efficacy of meropenem and INR. The time in the therapeutic range (TTR) can reflect the quality of anticoagulation of warfarin in clinical practice. A study predicted that a TTR of 58% would be considered as beneficial for anticoagulation treatment [13]. In this case, a TTR of 35.7% according to the Rosendaal method [14] was relatively low, indicating the poor anticoagulation effect of warfarin, which could be caused by the accompanied infectious condition.

In this case, under the suggestion of personal medication, the expected anticoagulation effect was not achieved until meropenem was used along with warfarin. The anticoagulant effect of warfarin and the side effects of bleeding are mainly caused by many factors such as disease, dietary vitamin K intake, genetic factors, and drugs [5,15–18]. Other factors that could affect the efficacy and pharmacokinetics of warfarin except for infection could be excluded, and no data indicate that meropenem is related to the enhanced anticoagulant effect of warfarin. Therefore, the interaction between *E. coli* infection and warfarin was speculated to inhibit the anticoagulant effect of warfarin.

Vitamin K antagonists such as warfarin have been applied in the anticoagulation treatment of thrombosis diseases for decades. In recent years, direct oral anticoagulants (DOACs), including rivaroxaban, edoxaban, apixaban, and dabigatran, have been developed to offer efficient anticoagulation [19–23], and have been increasingly applied in the clinical management of thrombosis diseases. However, in patients with severe renal insufficiency or inability to cover the costs of DOACs, warfarin remains a critical option for treatment. In our country, due to the relatively high price of DOACs, warfarin is still the first choice of anticoagulation treatment and DOACs would be applied in the conditions of warfarin intolerance.

Warfarin acts as an anticoagulant by inhibiting the activity of VKOR in human bodies. VKOR homologues are also found in some bacteria. It is reported that warfarin can inhibit the growth of customarily conjugated mycobacteria after *VKOR* knockout in mycobacteria, and inhibit bacterial disulfide bond formation [24]. Thus, the homolog of bacterial *VKOR* may be the target of warfarin. Disulfide bond formation protein B (DsbB) is necessary for the formation of disulfide bonds in *E. coli*. Follow-up studies have shown that DsbB and VKOR expressed by *E. coli* have no apparent homology at the primary sequence level. Still, both can catalyze the formation of disulfide bonds, a structure of the bacterial homolog of vitamin K epoxide reductase [25]. Therefore, we speculated that the DsbB protein expressed in *E. coli* has an antagonistic effect similar to that of VKORC1. During warfarin use, the DsbB protein expressed by *E. coli* in the patient’s blood may have interacted with warfarin, resulting in a low INR in the patient.

**Figure A1:**
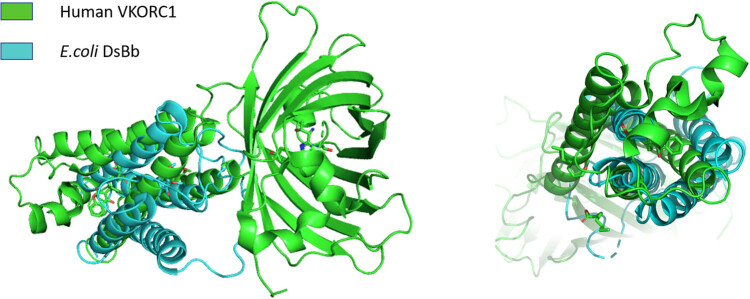
Three-dimensional structure of VKORC1 and DsBb. The similarity of the three-dimensional structure between DsbB and VKORC1 is low. The whole genome of the clinical *E. coli* isolate obtained from the patient’s blood culture was sequenced, and the result showed a *DsbB* gene on the genome.

To further explore whether the interaction exist between warfarin and *E. coli*, the whole genome of the clinical isolates of *E. coli* obtained from the patient’s blood culture was sequenced. After the *DsbB* gene was sequenced, the protein structures of DsbB and VKORC1 were predicted. Although they both possess oxidoreductase activity, the result shows that the similarity of the three-dimensional structure between DsbB and VKORC1 is low (Figure A1). Further experiments like the competitive binding of the VKORC1 protein and *E. coli* with warfarin are needed to directly verify whether *E. coli* can interact with warfarin. Simultaneously, substances interacting with *E. coli* can be determined by combining a proteomics approach.

To conclude, this case was the first to suggest that *E. coli* infection may interact with warfarin. The use of warfarin is complicated because of its narrow treatment window, considerable individual differentiation, and interaction with many foods, drugs, and diseases, thus requiring close monitoring. So when patients are in thromboembolic conditions complicated by infection, we should closely monitor the INR to apply the proper dose of warfarin and to prevent the influence of pathogenic microorganisms on warfarin.
